# Characterization of a complex rearrangement involving duplication and deletion of 9p in an infant with craniofacial dysmorphism and cardiac anomalies

**DOI:** 10.1186/1755-8166-5-31

**Published:** 2012-07-09

**Authors:** Daniel L Di Bartolo, Mohamed El Naggar, Renius Owen, Trilochan Sahoo, Fred Gilbert, Venkat R Pulijaal, Susan Mathew

**Affiliations:** 1 Department of Pathology and Laboratory Medicine, Weill Cornell Medical College/NewYork-Presbyterian Hospital, New York, NY, USA; 2 Quest Diagnostics Nichols Institute, San Juan Capistrano, CA, USA; 3 Department of Pediatrics, Weill Cornell Medical College, New York, NY, USA

**Keywords:** Concomitant deletion and duplication of 9p, Deletion 9p Syndrome, Duplication 9p Syndrome, Craniofacial dysmorphism, Cardiac anomalies

## Abstract

Partial duplication and partial deletion of the short arm of chromosome 9 have each been reported in the literature as clinically recognizable syndromes. We present clinical, cytogenetic, and molecular findings on a five-week-old female infant with concomitant duplication and terminal deletion of the short arm of chromosome 9. To our knowledge ten such cases have previously been reported. Conventional cytogenetic analysis identified additional material on chromosome 9 at band p23. FISH analysis aided in determining the additional material consisted of an inverted duplication with a terminal deletion of the short arm. Microarray analysis confirmed this interpretation and further characterized the abnormality as a duplication of about 32.7 Mb, from 9p23 to 9p11.2, and a terminal deletion of about 11.5 Mb, from 9p24.3 to 9p23. The infant displayed characteristic features of Duplication 9p Syndrome (hypotonia, bulbous nose, single transverse palmar crease, cranial anomalies), as well as features associated with Deletion 9p Syndrome (flat nasal bridge, long philtrum, cardiac anomalies) despite the deletion being distal to the reported critical region for this syndrome. This case suggests that there are genes or regulatory elements that lie outside of the reported critical region responsible for certain phenotypic features associated with Deletion 9p Syndrome. It also underscores the importance of utilizing array technology to precisely define abnormalities involving the short arm of 9p in order to further refine genotype/phenotype associations and to identify additional cases of duplication/deletion.

## Background

Rearrangements involving the distal region of 9p, particularly p22-p24, have been well described in the literature and are associated with either Duplication 9p Syndrome or Deletion 9p Syndrome (OMIM 158170) [1-5]. Few cases, however, have been reported with concurrent duplication and terminal deletion of 9p [5-14]. Here we present another such case and compare our clinical and cytogenetic findings with those in the literature.

### Clinical report

The patient is the third child of a healthy 19-year-old woman of African-American and Hispanic descent. She was born preterm at 36 weeks gestation. At birth, she weighed 2.7 kg; her head circumference was 33.5 cm and length was 48 cm (all around the 10^th^ percentile). She was evaluated by pediatric cardiology at three days of life because of a heart murmur. An echocardiogram, at that time, revealed a small perimembraneous ventricular septal defect, moderate patent ductus arteriosus, and a moderate to large secundum atrial septal defect. She initially had hypothermia and hypoglycemia, however, these conditions resolved quickly. She also exhibited hypotonia and intermittent tongue thrusting.

Notable findings on physical examination at five weeks of age included wide-open anterior fontanelle and sagittal suture, prominent nose, flat nasal bridge, long grooved philtrum, thin lips, extra skin at the back of the neck, and a single transverse palmar crease on the left hand. External genitalia were that of a normal female.

## Results

### Cytogenetics and fluorescence *in situ* hybridization (FISH)

**Figure 1:**
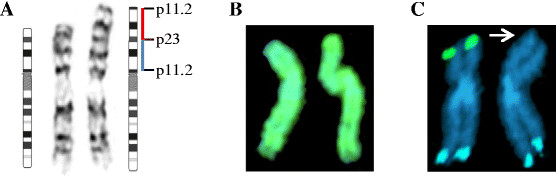
**Identification and characterization of the 9p abnormality by conventional cytogenetics and FISH analysis.** (**A**) G-banded chromosomes 9 with der(9)(pter→p24.3::p11.2→p23::p23→qter). Ideogram showing the region that is duplicated, p11.2-p23 (blue bar), and inverted (red bar). (**B**) FISH analysis using WCP 9 and (**C**) subtelomeric probes 305J7T7 for 9p (green) and LSI 9q34 for 9q (aqua). Arrow indicates terminal deletion of 9p.

Cytogenetic analysis identified additional material on one of the homologs of chromosome 9 at band p23 (Figure 1A). FISH analysis using whole chromosome 9 painting probe, WCP 9, revealed the extra material was comprised entirely of material from chromosome 9 (Figure 1B). Additionally, FISH identified a terminal deletion of 9p using the 9p subtelomeric probe, 305J7T7 (Figure 1C). Based on both conventional cytogenetics and FISH analysis, the derivative chromosome 9 was determined to have an inverted duplication of the region between bands p11.2 and p23 as well as a concurrent deletion of the terminal pter-p23 segment. We were unable to determine whether this abnormality was inherited or *de novo* as parental blood was unavailable.

### Microarray analysis

**Figure 2:**
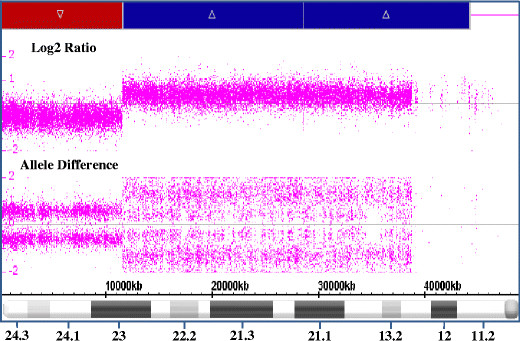
**Characterization of the breakpoints involved in the duplication and deletion of 9p using the Affymetrix Genome-Wide Human SNP Array 6.0.** The plot shows chromosome 9 log2 test over reference ratio and allelic difference (Y-axis) plotted against the Mb position from pter to the centromere (X-axis). A significant terminal loss of approximately 11.5 Mb, from 9pter to 9p23, and an adjacent duplication of approximately 32.7 Mb, from 9p23 to 9p11.2, were identified.

Affymetrix Genome-Wide Human SNP Array 6.0 confirmed the apparent interstitial duplication and distal deletion on the short arm of chromosome 9 (Figure 2). The duplication was found to span approximately 32.7 Mb, from 11,576,113 bp (9p23), to 44,244,868 (9p11.2). The proximal deletion breakpoint occurred between genomic probe positions 11,575,785 and 11,576,113 (9p23), with the deletion encompassing the region distal to the breakpoint, indicating that genes including and distal to *PTPRD* are deleted and those proximal to this break on the p arm are duplicated. The final karyotype after molecular characterization was: 46,XX,der(9)(pter→p24.3::p11.2→p23::p23→qter).arrcgh9p24.3p23(37,746-11,575,785)x1,9p23p11.2(11,576,113-44,244,868)x3.

## Discussion

**Figure 3:**
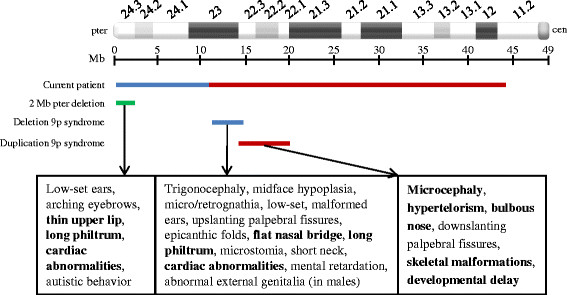
**Comparison of deletion and duplication in the present case with the previously reported 2 Mb terminal deletion**[10]**, the proposed critical region in Deletion 9p Syndrome, and the region associated with Duplication 9p Syndrome.** Clinical features associated with each region are indicated. Features in the present case that correspond to the deleted or duplicated regions are shown in bold.

Partial duplication of 9p is one of the most commonly detected autosomal structural abnormalities in live-borns [15,16]. Despite variability in size of the duplicated segment, patients with partial duplication of 9p display considerable phenotypic similarity. Characteristic features of this syndrome, which include microcephaly, hypertelorism, upslanting palpebral fissures, broad nasal root with bulbous nasal tip, downturned corners of the mouth, anomalous ears, single palmar crease, skeletal malformations, hypotonia, and developmental delay, are thought to result from duplication of genes lying within 9p22 [15,16]. The current case has a duplication of approximately 32.7 Mb, from 9p23 to 9p11.2, and displays some of the cardinal features of Duplication 9p Syndrome (Figure 3).

Deletion 9p syndrome is a well described clinical entity with over 80 cases reported in the literature. Phenotypic features include mental retardation, trigonocephaly, midface hypoplasia, flat nasal bridge, long philtrum, microstomia, micro/retrognathia, cardiac anomalies, and genital anomalies in males. Work from several groups has narrowed down the critical region to p22.2-p23, a 3.5 Mb region between 11.4-14.9 Mb from the 9p telomere [4,9,17,18]. More recently, Swinkels et al. proposed refining the critical region to approximately 300 kb within band 9p22.3 [5].

The deletion in the present case lies distal to the critical region proposed by these groups. Nonetheless, our patient displays features associated with Deletion 9p Syndrome such as long philtrum, flat nasal bridge, and heart murmurs/defects. Similar reports of patients with distal terminal deletions exhibiting features of Deletion 9p Syndrome have also been reported [14,19]. Recently, Hauge et al. identified a minimal 9p terminal deletion of 2 Mb, distal to the previously described critical region, in 5 patients who manifested features common to Deletion 9p Syndrome [10]. Figure 3 depicts the proposed critical region for Deletion 9p Syndrome, the 2 Mb minimally deleted region [10], the deleted region in the present case, and the clinical features associated with each. Our report, in addition to similar reports [10,14,19], submits that deletion of genes more distal to the previously described critical region contributes to features commonly associated with Deletion 9p Syndrome (Figure 3) and that further refinement to the genotype/phenotype correlation for this syndrome is needed.

One of the major features of Deletion 9p Syndrome, trigonocephaly, was absent in the present case. Previous reports have mapped trigonocephaly to a region in p22.3 between 14.7 and 15.1 Mb. Two genes that lie within this region, *FREM1* and *CER1*, have been implicated as candidate genes [18,20]. *FREM1* mutations exhibit variable penetrance and expressivity, suggesting that other factors, possibly mutations or deletions of additional genes or regulatory elements, contribute to the phenotype. Features of trigonocephaly have also been reported in patients with deletions that lie distal to *FREM1*[4,10,19], further suggesting that other genes may be involved.

In contrast to trigonocephaly, the patient in the present study exhibited delayed closure of the anterior fontanelle and sagittal suture. This feature is occasionally seen in patients with Duplication 9p Syndrome. Interestingly, the duplicated region in our case coincides with the deleted region commonly associated with trigonocephaly. It is possible that a dosage sensitive gene (or genes), possibly *FREM1* and/or *CER1*, is involved in the development and/or closure of skeletal plates.

Of the genes that fall within the deleted region in our patient (Additional file 1: Table  S1), several are associated with a particular phenotype. Deletion of *FOXD4* is associated with speech and language delays [10], and mutations in a related forkhead box gene, *FOXP2*, cause linguistic impairment and difficulties with motor coordination [21]. Haploinsufficiency of *DMRT12*, and *3* may result in ambiguous external genitalia, particularly in karyotypic males, as well as gonadal dysgenesis [7,22,23]. Disruption of *DOCK8* was identified in patients with mental retardation and/or seizures [10,24]. Similarly, mutations in *VLDR1* are associated with cognitive impairment, cerebellar ataxia, and seizures [25]. Our patient exhibited intermittent tongue thrusting, however, whether it is associated with mental retardation or epilepsy is difficult to assess due to her age. Deletions within the paternal *KANK1* gene have been identified in several children with cerebral palsy [26]. The significance of the *KANK1* deletion in our patient is unknown as the parental origin of the 9p deletion/duplication was not able to be determined.

**Figure 4:**
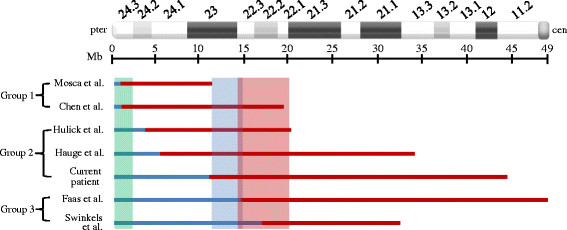
**Comparison of all reported cases of deletion and duplication of 9p for which microarray analysis was performed.** Shaded boxes represent the 2 Mb terminally deleted region (green), the proposed critical region for Deletion 9p Syndrome (blue), and the region associated with Duplication 9p Syndrome (red). Group 1 includes cases with deletions below 2 Mb, Group 2 comprises cases with deletions covering the 2 Mb terminal region, and Group 3 includes cases with deletions encompassing the proposed critical region for Deletion 9p Syndrome (Group 3).

To our knowledge, 10 cases with both a proximal duplication and a terminal deletion of 9p have previously been reported. Comparison of the deleted and duplicated regions in the current case and reported cases for which microarray analysis had been performed [5,9-12,14] is given in Figure 4. Deletions are placed into three groups: Group 1 (two cases) with terminal deletions of less than 2 Mb, Group 2 (three cases) with terminal deletions of 3.7-11.5 Mb, encompassing the 2 Mb minimally deleted terminal region of 9p identified by Hauge et al. [10], and Group 3 (two cases) with larger deletions of 14.8-17.3 Mb, which include the Deletion 9p Syndrome critical region. The size of the duplication varied between cases with most encompassing the p22 region. Clinical features varied among cases (Table 1), owing to the different sizes and locations of the deletion and duplication; nevertheless, they could be divided into 3 categories: those associated with a 2 Mb terminal deletion of 9p, those related to Deletion 9p Syndrome, and those common to Duplication 9p Syndrome.

**Table 1:** Clinical features in patients with 9p duplication and deletion

Authors	Congenital anomalies
Mosca et al.^a^(12 years)	*synophyrs*, *upslanting palpebral fissures*, *short neck*, ***mental retardation***, **microcephaly**, large ears, hypotonic face, low hairline, Polymicrogyria
Chen et al. (22 wks gestation)	thin lips,*narrow forehead with metopic ridging*, *long, thin eyebrows with mildsynophyrs, short neck*, ventriculomegaly
Hulick et al. (4 months)	thin upper lip,*mild micrognathia*, *low-set ears*, *microstomia*, ***hypertelorism***, **bulbous nose**, **cleft palate**, **5**^**th**^ **digit bilateral clinodactyly**, **dystrophic nails**, absent uvula, deep-set eyes, large palpebral fissures, difficulty feeding, growth delay
Hauge et al. (7 years)	thin upper lip,*low-set, posteriorly rotated ears*, *hypotonia*, ***mild hypertelorism,developmental delay***, **microcephaly**, **long palms**, **5**^**th**^**digit bilateral clinobrachydactyly**, peg-shaped teeth, low-set eyebrows, smooth philtrum, mild hearing loss
Current case (5 weeks)	*thin lips,cardiac murmur/deficit,long, grooved philtrum,flat nasal bridge*, *hypotonia*, **wide-open anterior fontanelle and sagittal suture**, **microcephaly**, **bulbous nose**, **single palmar crease**, extra skin at nape
Faas et al. (2 years)	thin lips,*long*, *prominent philtrum,cardiac murmur/deficit,metopic ridge,high,narrow forehead with bitemporal narrowing,retrognathia,narrow,upslanting palpebral fissures,anteverted nostrils,microstomia,short neck,widely space nipples,****hypertelorism***, **narrow palate,low-set ears,long fingers and toes,small nails,clinodactyly of 4**^**th**^**and 5**^**th**^**digits,** blue sclerae
Swinkels et al. (10 years)	thin upper lip,*long philtrum*, *cardiac murmur/deficit,trigonocephaly*, *micrognathia*, *midfacehypoplasia*, *small palpebral fissures*, *epicanthic folds*, *posteriorly rotated, low-set ears, flat nasal bridge, anteverted nostrils*, *short neck*, *widely spaced nipples*, *omphalocele*, ***developmental delay***, ***hypertelorism***, **highly arched**, **narrow palate**, **tapering fingers, scoliosis, flat feet**

We report the eleventh case involving partial duplication of 9p with a concomitant cryptic terminal deletion. Similar cases have likely been missed in the past due to the sole use of conventional cytogenetics which has undoubtedly confounded genotype/phenotype correlations, underscoring the importance of using molecular techniques to identify these often small, however, clinically significant terminal deletions. The use of microarray technology is critical for the proper identification and precise molecular characterization of these abnormalities and will aid in better defining critical regions, facilitating more accurate genotype/phenotype correlations, and potentially describing new clinical syndromes, such as 9p Deletion/Duplication Syndrome.

## Materials and methods

### Cytogenetics and FISH

Peripheral blood from the patient was cultured for 72 hr in the presence of phytohemagglutinin. Metaphase spread preparations and GTG-banding were performed according to standard methods. FISH analysis was performed using WCP 9 and subtelomeric probes, 305J7T7 for 9p (green) and LSI 9q34 for 9q (aqua) (Vysis/Abbott Molecular Inc., Des Plaines, IL).

### Molecular karyotyping

SNP array was performed using the Affymetrix Genome-Wide Human SNP Array 6.0, which contains 906,600 single nucleotide polymorphism probes (SNPs) and over 946,000 probes for copy number (Affymetrix, Santa Clara, CA, USA). Manufacturer-provided protocols for experimentation (http://media.affymetrix.com/support/downloads/manuals/) and analysis (Chromosome Analysis Suite (ChAS) software package, version 1.2) were followed. Genomic positions are given as mapped to the GRCh37/hg19 genome build.

## Consent

Written informed consent for this cytogenetic study was obtained from a parent of the patient. A copy of the written consent is available for review by the Editor-in-Chief of this journal.

## Competing interests

The authors declare they have no competing interests.

## Authors’ contributions

DD analyzed conventional and molecular cytogenetic findings, collected relevant data, and drafted the paper. ME, RO, and TS performed the microarray studies and analysis. FG performed clinical examination of the patient and provided insight into the manuscript. VP and SM supervised the conventional and molecular cytogenetic analysis. SM provided insight into the manuscript. All of the authors read and approved the final manuscript.

## Supplementary Material

Additional file 1 **Table S1.** Deleted genes in order from 9pter to p23. Click here for file
