# Supplementary material for: Characterization of a complex rearrangement involving duplication and deletion of 9p in an infant with craniofacial dysmorphism and cardiac anomalies
Source: Mol Cytogenet. 2012 Jul 9;5:31. doi: 10.1186/1755-8166-5-31 (PMC3419606; doi:10.1186/1755-8166-5-31)
Supplement: Additional file 1 — Table S1. Deleted genes in order from 9pter to p23. [file 1755-8166-5-31-S1.rtf]

Supplemental Table 1. Deleted genes in order from 9pter to p23

AY343892, AY343902, FOXD4, CBWD1, C9orf66, DOCK8, KANK1, DMRT1, DMRT3, DMRT2, SMRCA2, FLJ35024, VLDR, KCNV2, KIAA0020, Mir-548, RFX3, BC069756, GLIS3, Mir-320, SLC1A1, C9orf68, PPAPDC2, CD37L1, AK3, RCL1, MIR101-2, AK021739, JAK2, TRNA-Gln, INSL6, INSL4, RLN2, RLN1, C9orf46, CD274, PDCD1LG2, KIAA1432, ERMP1, MLANA, MIR4665, KIAA2026, RANBP6, IL33, TPD52L3, UHRF2, GLDC, KDM4C, DQ580140, Mir-584, C9orf123, PTPRD
